# Impacts of daily household activities on indoor particulate and NO_2_ concentrations; a case study from oxford UK

**DOI:** 10.1016/j.heliyon.2024.e34210

**Published:** 2024-07-05

**Authors:** Ajit Singh, Suzanne E. Bartington, Pedro Abreu, Ruth Anderson, Nicole Cowell, Felix C.P. Leach

**Affiliations:** a Institute of Applied Health Research, University of Birmingham, Edgbaston Park Road, Birmingham, B15 2TT, UK; b School of Geography, Earth and Environmental Sciences, University of Birmingham, Edgbaston Park Road, Birmingham, B15 2TT, UK; c Oxford City Council, St Aldates Chambers, 109 St Aldates, Oxford, OX1 1DS, UK; d Oxfordshire County Council, County Hall, New Road, Oxford, OX1 1ND, UK; e Centre for Environmental Policy, Imperial College London, Weeks Building, 16-18 Prince's Garden, London SW7 1NE, UK; f Department for Engineering Science, University of Oxford, Parks Road, Oxford, OX1 3PJ, UK

**Keywords:** Indoor air quality, PM, NO_2_, Low-cost sensor, COVID-19

## Abstract

This study explores indoor air pollutant (PM_1_, PM_2.5_ and NO_2_) concentrations over a 15-week period during the COVID-19 pandemic in a typical suburban household in Oxford, UK. A multi-room intensive monitoring study was conducted in a single dwelling using 10 air quality sensors measuring real-time pollutant concentrations at 10 second intervals to assess temporal and spatial variability in PM_1_, PM_2.5_ and NO_2_ concentrations, identify pollution-prone areas, and investigate the impact of residents' activities on indoor air quality. Significant spatial variations in PM concentrations were observed within the study dwelling, with highest hourly concentrations (769.0 & 300.9 μg m^−3^ for PM_2.5_, and PM_1_, respectively) observed in the upstairs study room, which had poor ventilation. Cooking activities were identified as a major contributor to indoor particulate pollution, with peak concentrations aligning with cooking events. Indoor NO_2_ levels were typically higher than outdoor levels, particularly in the kitchen where a gas-cooking appliance was used. There was no significant association observed between outdoor and indoor PM concentrations; however, a clear correlation was evident between kitchen PM emissions and indoor levels. Similarly, outdoor NO_2_ had a limited influence on indoor air quality compared to kitchen activities. Indoor sources were found to dominate for both PM and NO_2_, with higher Indoor/Outdoor (I/O) ratios observed in the upstairs bedroom and the kitchen. Overall, our findings highlight the contribution of indoor air pollutant sources and domestic activities to indoor air pollution exposure, notably during the COVID-19 pandemic when people were typically spending more time in domestic settings. Our novel findings, which suggest high levels of pollutant concentrations in upstairs (first floor) rooms, underscore the necessity for targeted interventions. These interventions include the implementation of source control measures, effective ventilation strategies and occupant education for behaviour change, all aimed at improving indoor air quality and promoting healthier living environments.

## Highlights

• Nitrogen dioxide (NO_2_) and Particulate Matter (PM) variations in a suburban household during COVID-19 are examined.
• PM levels varied among rooms, with gas cooking the main indoor pollutant source.
• Indoor NO_2_, particularly kitchen consistently surpassed outdoor levels & World Health Organization (WHO) limits.
• Poor ventilation in well-insulated homes poses indoor air quality challenges.
• Study shows need to reduce indoor pollutant levels within typical UK suburban homes.

## Graphical abstract

**Figure:**
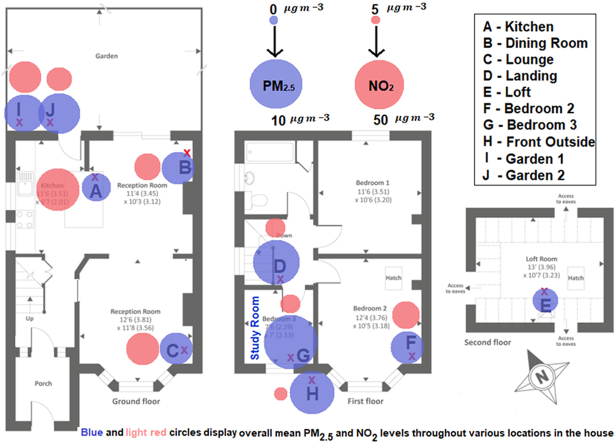

## 1 Introduction

Air pollution from both indoor and outdoor sources is a major environmental concern and epidemiological evidence associates exposure to pollutants with a wide range of adverse health impacts [[1], [2], [3]], including cancer, respiratory and cardiovascular diseases and increased risk of premature mortality [[4], [5], [6]]. The main pollutants of public health concern in the UK are particulate matter (PM) and nitrogen oxides (NO_x_). Particles with aerodynamic diameters less than 1 μm (PM_1_) and 2.5 μm (PM_2.5_) are of great importance in indoor household studies due to their fine particle sizes. These particle sizes are associated with significant health impacts, affecting the human respiratory, cardiovascular, metabolic and neurological systems and all-cause mortality [[7], [8], [9], [10]]. Further, nitrogen dioxide (NO_2_) also holds significance indoors as it is a respiratory irritant, associated with the development and increased severity of childhood asthma [[11], [12], [13]].

The complexity of air pollution exposure assessment for health impact studies is in part due to its spatial heterogeneity, where air pollution varies across different locations, microenvironments and spaces [14,15] which vary for different air pollutants [15,16]. Air pollution also exhibits substantive temporal variability, with peak concentrations associated with high magnitude short-term exposure [17,18]. Whilst it is important to understand how air pollution varies between types of location and over time, in epidemiological studies it is also important to both indoor and outdoor environments notably locations where significant numbers of people are to be found, or where people spend significant amounts of time. Ambient air pollution exposure has traditionally been a major focus of research efforts, yet humans in industrialised settings typically spend 60–90 % of their time indoors [19]. Further, during the COVID-19 pandemic successive emergency public health restrictions led to a greater proportion of time spent in indoor environments, due to restrictions on work and leisure activities [20].

Recent studies suggest indoor air pollutant concentrations in UK domestic settings can regularly exceed WHO Global Air Quality Guidelines and peak air pollutant concentrations in indoor environments can be higher than those in outdoor environments [[21], [22], [23]].

Higher PM concentrations in indoor compared to outdoor environments have been associated with specific sources of indoor particulate in domestic homes. Examples of this include cooking, smoking, the use of indoor combustion appliances such as gas heaters or open stoves, as well as insufficient ventilation, among others [22,[24], [25], [26]]. However, while there are a number of studies that have assessed indoor air pollution, monitoring has generally been limited to only one or two locations within the property [[27], [28], [29], [30], [31], [32]]. More recent studies which have undertaken assessment in multiple domestic rooms have been limited to PM only [22,33]. Therefore, although particulate matter (PM) and gaseous pollutants (e.g. NO and NO_2_, etc.) are recognised to be strongly related to domestic activities, with potentially harmful indoor concentrations, few studies have investigated multi pollutant concentrations in multiple rooms within a domestic setting. Such studies would be of value for future exposure assessment studies.

Air quality sensors are generally small, portable devices that use a variety of technologies to detect and measure air pollutants, such as PM, NO_2_ and ozone [34]. Sensors are typically much less expensive than traditional grade air quality monitoring equipment and can be deployed in a much wider range of locations, including indoor environments. Over the past few years, the accessibility and range of air quality sensor technologies have increased, which has revolutionized capabilities for monitoring and evaluation in both outdoor and indoor settings [[35], [36], [37], [38], [39], [40], [41]]. However, factors such as temperature, relative humidity, and particle composition can affect sensor performance [42].

Expanding upon previously published work that examined the influence of COVID-19 restrictions on outdoor air quality using a validated low-cost sensor network in Oxford [41], this study further explores changes in indoor air quality in relation to domestic activities, in the context of the COVID-19 pandemic. The current study deployed a network of air quality sensors (South Coast Science, Praxis/Urban units) to assess spatial and temporal variations in PM_1_, PM_2.5_ and NO_2_ concentrations across multiple rooms in a single residential property during a period of COVID-19 restrictions. By using a network of air quality sensors deployed within a residential property, this study aims to assess comprehensively the impact of domestic activities on indoor air quality during a period of national restrictions, with potential implications for public health. Specific aims were to:

a) Characterise and quantify the temporal and spatial air quality variations within a single dwelling setting and identify pollution-prone areas.
b) Evaluate the impact of domestic activities and different sources on PM and NO_2_ pollution levels.
c) Establish correlations between indoor and outdoor air quality parameters to understand the influence of outdoor pollution sources on indoor environments.

## 2 Methods

### 2.1 Study setting

This study was undertaken in a single suburban domestic setting located in a residential area of Oxford, UK; a city with population of ∼150,000 and recognised air quality challenges [43]. The whole city was declared an Air Quality Management Area in 2010. The study property is a semi-detached dwelling, constructed in 1930, with garden located in a cul-de-sac approximately 2 km from the city centre and 360 m from the nearest major road. The property has an internal floor area of approximately 86.2 m^2^, three bedrooms (Bedrooms 1–3), one bathroom, one kitchen, dining room, lounge, and garden. The house is of a very common design in Oxford and is broadly reflective of suburban properties in other UK cities. Fig. S1 shows the floor plan of house property. The property is fitted with modern, double-glazed, well insulated external doors and windows and the chimneys have been sealed. A well-maintained gas boiler (Glowworm Flexicom 24CX), ventilating outside the propery is located in the bathroom on the first floor. The kitchen is fitted with a four-ring gas hob (Electrolux EGH6343BOX) and an electric oven. A recirculating ventilation hood (i.e. not ventilating outside) is fitted above the hob (Electrolux EFC60151X). A printer (HP LaserJet 1022n) is located in Bedroom 3, which was used as a study and not as a bedroom. At the time of this work, the property had two residents, both of whom were non-smokers. Both residents were working at home during this stage of the COVID-19 pandemic. There were no additional visitors to the household during the study period in accordance with the public health restrictions in force at the time.

### 2.2 Data collection: air quality sensors

This study deployed 10 (3 outdoors, 7 indoors) South Coast Science Praxis/Urban air quality monitors comprising Alphasense Optical Particle Counter (OPC–N3), Alphasense NO_2_–B43F Nitrogen Dioxide sensor and Sensirion SHT45 sensor to measure PM mass concentration (PM_1_ and PM_2.5_), NO_2_ concentration and meteorology (relative humidity and temperature), respectively. Previous studies have shown that the data collected from these sensors are high quality and meet with established standards for this type of measurement – with values within ±2.6 ppb of the reference method for NO_2_, ±4.4 μg m^−3^ for PM_10_ and ±2.7 μg m^−3^ for PM_2.5_ [41,44].

The OPC-N3 is a high-performance optical particle counter that can detect and measure the concentration of airborne particles. It is commonly used for indoor and outdoor air quality monitoring. OPC-N3 sensors are small in size (dimensions 75 × 60 × 65 mm) and weight (about 150 g) and have relatively low power requirements (175 mA), which allows their operation in a variety of locations either with mains power or battery. OPC-N3 sensor measures the light scattered by individual particles carried through a laser beam, where these measurements are used to determine the particle size (based on Mie scattering theory) and particle number concentration. OPC-N3 sensor then calculates PM_1_, PM_2.5_ and PM_10_ from the particle size spectra (only PM_1_ and PM_2.5_ used in this work) and concentration data, assuming a particle density and refractive index (RI) [[45], [46], [47]]. The OPC-N3 measures particles in the size range of 0.35–40 μm, with a maximum particle count of 10,000 per second.

Alphasense NO_2_–B43F nitrogen dioxide sensors are portable, light in weight (<13 g) and easy to use. The lightweight design of the Alphasense NO_2_–B43F sensors makes them easy to carry around and deploy in different settings, which is particularly useful for both indoor and outdoor monitoring. Additionally, the user-friendly interface of these sensors makes them easy to operate, even for individuals who are not familiar with their operation. NO_2_–B43F sensor measures NO_2_ concertation using the electrochemical method (4-electrodes) based on electrochemical reactions [48]. NO_2_–B43F sensor provides high selectivity, low limit of detection with low power consumption and even can monitor small concentrations of NO_2_ levels (ppb level).

Sensirion SHT45 sensors are also mounted along with air quality sensors to monitor relative humidity and air temperature. This is the perfect fit for mobile and battery-driven applications due to very low power requirement. The Sensirion SHT45 sensor is an excellent addition to air quality monitoring systems, as it provides valuable information about the meteorological conditions that can impact indoor home air quality.

### 2.3 Sampling design

10 SCS Praxis units were deployed across the property to undertake monitoring for a three-month period during the COVID-19 period (April 22, 2020 to July 22, 2020). This period largely coincided with the first national ‘lockdown’ (23 March–15 June 2020) [49] Particle (PM_1_ and PM_2.5_) and gas (NO_2_) pollutant concentrations and relative humidity and temperature, were sampled at a 10-s time resolution (which was then down-sampled to 1-min intervals) at 10 locations across the property, selected to reflect a broad range of domestic activity locations which included seven indoor and three outdoor locations (Table 1). All times noted in this work are Greenwich Mean Time (GMT) which was 1 h behind British Summer Time (BST) – local time – at the time of the study. Indoor and outdoor air samples were taken simultaneously at the study location over the study period. Data for temperature and relative humidity were also recorded at 1 min time intervals at each sampling location. In addition, hourly NO_2_ and PM_2.5_ were also obtained at the nearest Automated Urban and Rural Network (AURN) urban background site (St Ebbe's, site number - UKA00518), located 2.4 km South West of the study dwelling, for the duration of the study period [50].

**Table 1:** Description of deployed air quality sensors, their location, potential pollution sources and ventilation arrangements.

Location	Sensor reference	Primary activities/potential particulate and NO_2_ sources	Ventilation	Sampling location	Sensor height (m above floor level)
Kitchen	A	Cooking, cleaning	Window, door, recirculating exhaust fan	On countertop	1.05
Dining Room	B	Cooking, cleaning	Window, door	Table	0.75
Lounge	C	Cleaning	Window	Bookshelf	0.9
Landing	D	Cleaning, (gas) boiler	Window	Table	0.7
Loft	E	None	Window	Floor	0
Bedroom 2 (upstairs)	F	Cleaning	Window	Desk	0.75
Bedroom 3 (upstairs) – used as a study	G	Cleaning, printing	None	Filing cabinet	1.05
Front outside	H	Outdoor	N/A	On front wall of house facing road (8 m from road edge)	4.4 (above ground)
Garden 1	I	Outdoor	N/A	On floor under rain shelter	0
Garden 2	J	Outdoor	N/A	Under rain shelter	1.95

### 2.4 Domestic activity log

In order comprehensively to capture relevant domestic activities that could contribute to particulate or gaseous air pollutant emissions, a manual activity log was meticulously maintained by the residents participating in the study. This log documented specific activities undertaken within the residential setting that could potentially influence indoor air quality. The data collection process involved residents noting down each activity along with relevant details such as the type of activity, its duration, and any notable emissions or pollutant sources associated with it (see Table S1). This meticulous record-keeping allowed for a granular understanding of the various indoor activities that might impact the indoor air quality environment.

## 3 Results and discussion

### 3.1 Particulate pollution concentrations

**Fig. 1:**
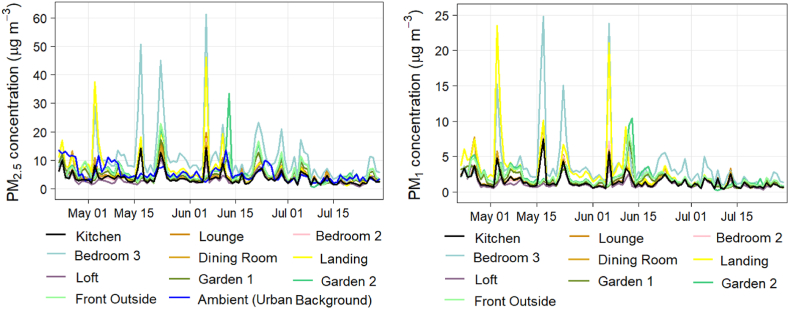
Daily variations of PM_2.5_ and PM_1_ mass concentrations observed at multiple locations within the study property.

Fig. 1 shows the daily patterns of particle mass concentrations at various locations within and outside the residential property, comparing them to the nearest urban background monitoring location. In general, overall study mean PM_2.5_ concentration values were almost the same between the front exterior of the property (5.72 μg m^−3^) and the urban background site (5.32 μg m^−3^); however, relatively higher peak values were noted at the front exterior of the property compared to the urban background site. The overall study indoor mean PM_2.5_ concentrations measured inside the dwelling were also found to be similar (5.36 μg m^−3^) when compared to the urban background site (5.32 μg m^−3^).

***PM variations in microenvironments at the study dwelling*** – Measured indoor PM concentrations varied substantially over the study (see Fig. S2 and Table 2). The highest hourly peaks of indoor PM_1_ and PM_2.5_ concentrations were observed at upstairs Bedroom 3 location (300.99 μg m^−3^ and 769.07 μg m^−3^, respectively) while lowest concentrations were observed in the loft (43.18 μg m^−3^ and 104.94 μg m^−3^, respectively). Similar temporal patterns were observed in the kitchen and other adjacent rooms strongly suggesting that cooking activities were the major source of indoor particulate matter emissions. Peak PM concentrations corresponded closely with household (cooking and other) activity patterns, with similar temporal patterns observed in the kitchen and adjacent locations. However, PM levels downstairs were relatively lower in the kitchen compared to the Bedroom 3 location upstairs. Cooking in the oven was the most common type of cooking method, with 55.5 % of all cooking recorded via the oven, 36.0 % via the gas hob and the rest by other appliances (i.e. kettle, toaster and microwave etc.). It was noted that the highest hourly PM peak episodes occurred when frying and using the hob (see Table S1).

**Table 2:** Hourly mean PM_2.5_, PM_1_ and NO_2_ concentrations and summary statistics.

Location	PM_2.5_ (mean) in μg m^−3^	PM_2.5_ (range) in μg m^−3^	PM_1_ (mean) in μg m^−3^	PM_1_ (range) in μg m^−3^	NO_2_ (mean) in μg m^−3^	NO_2_ (range) in μg m^−3^
**Kitchen (A)**	3.87	0.26–127.13	1.50	0.10–52.10	45.58	5.15–221.07
**Dining room (B)**	4.41	0.26–148.53	1.63	0.11–55.91	23.91	1.91–184.04
**Lounge (C)**	4.65	0.26–222.94	1.68	0.10–75.99	29.18	0.84–238.87
**Landing (D)**	6.48	0.28–537.26	2.87	0.13–239.16	20.77	0.06–156.27
**Loft (E)**	3.21	0.24–104.94	1.15	0.10–43.18	11.50	0.05–95.20
**Bedroom 2 (F)**	4.22	0.32–172.70	1.62	0.14–71.73	24.52	0.24–105.97
**Bedroom 3 (G)**	10.68	0.57–769.07	3.67	0.21–300.99	17.93	0.03–152.2
**Front outside (H)**	5.72	0.54–111.52	1.96	0.21–39.15	8.87	0.05–36.23
**Garden 1 (I)**	4.83	0.47–69.38	1.68	0.15–29.63	29.42	0.04–75.70
**Garden 2 (J)**	6.00	0.10–86.40	2.10	0.10–66.38	22.71	0.07–86.47
**St. Ebbe's urban background (AURN)**	5.32	0.48–29.71	N/A	N/A	6.87	0.36–72.0

Throughout the study period, the daily (24-hr) mean PM_2.5_ levels at all the indoor locations within the property were observed occasionally to exceed (on 1.0–15.0 % of days) the World Health Organisation's 2021 PM_2.5_ health-based guideline level of 15 μg m^−3^ (daily average). Particularly, the highest exceedance was noted upstairs in Bedroom 3 (15.0 % or 15 days of the total sampling period), while the lowest was in the Loft (1.0 % or 1 days of the total sampling) (Table 3). When we compared Bedroom 3 PM concentrations to outdoors (both urban background and outside the home), we found that Bedroom 3 had approximately 78–123 % higher concentration levels than outdoor PM levels. PM levels in the kitchen were relatively lower (21–36 %) than those outdoors, while other indoor locations within the property had similar PM levels. The higher PM levels in Bedroom 3 upstairs are likely due to the fact that during the study period it was heavily used as a study – and both of the occupants of the house were working from home at the time. One work from home activity which undoubtedly contributed to the PM levels in this room was printing (see a later section). Of particular note is that this room is the smallest in the house and does not have external ventilation. When an event that contributed to a rise in PM (such as printing or vacuum cleaning) occurred, it took a long time for the PM to disperse, probably due to the lack of ventilation.

**Table 3:** Percentage of study days exceeding WHO Global Air Quality guidelines (24-h average) for PM_2.5_/NO_2_ concentrations during the study period.

Location	PM_2.5_ – Percentage of days exceeding the WHO Global Air Quality Guideline (24-hr average) value of 15 μg/m³ from the total study period (100 days).	NO_2_ – Percentage of days exceeding the WHO Global Air Quality Guideline (24-hr average) value of 25 μg/m³ from the total study period (100 days).
**Kitchen (A)**	2 %	100 %
**Dining room (B)**	2 %	35 %
**Lounge (C)**	3 %	80 %
**Landing (D)**	8 %	17 %
**Loft (E)**	3 %	3 %
**Bedroom 2 (F)**	2 %	50 %
**Bedroom 3 (G)**	15 %	5 %
**Front outside (H)**	3 %	0 %
**Garden 1 (I)**	1 %	78 %
**Garden 2 (J)**	3 %	28 %
**St. Ebbe's urban background (AURN)**	3 %	0 %

***Impact of time-of-day and day-of-the-week factors on indoor PM variations* –**Fig. S3 shows the time-of-day and day-of-week variation of PM_2.5_ and PM_1_ concentrations, respectively. The diurnal profile indicates that PM concentrations are relatively low at night and high during the day, particularly in the kitchen and Bedroom 3. This pattern is consistent with household activity patterns (see Supplementary Table S1 and Fig. S2), as people tend to be more active and engage in more activities during the day, which can generate higher levels of indoor PM air pollution and both residents were home throughout the day over the duration of this study. A clear contribution was made by cooking events, which occurred mostly during the morning, afternoon and evening cooking periods (breakfast, lunch, and dinner). These events typically created sharp spikes in PM concentrations indoors. Similar patterns observed between the kitchen and other indoor locations strongly suggest that cooking could be a major source of indoor PM, however upstairs Bedroom 3 had shown significant and sharp spikes compared to other indoor locations.

PM concentrations during weekends were higher than most weekdays corresponding to increased kitchen activities, while again the PM concentrations in Bedroom 3 upstairs was of a significantly higher magnitude. The reported higher levels of indoor PM over the weekend are linked to cooking and household activities (Supplementary Table S1 and Fig. S2)

### 3.2 NO_2_ pollutant concentrations

Overall, NO_2_ concentrations were found to be higher indoors than outdoors (including background levels). The typical hourly-averaged NO_2_ concentrations recorded in the kitchen were between 5.1 and 221.2 μg m^−3^, which are much higher than the typical hourly averaged NO_2_ concentrations recorded in the outdoor front area, which ranged from 0.05 to 36.23 μg m^−3^ (Fig. S4 and Table 2)

**Fig. 2:**
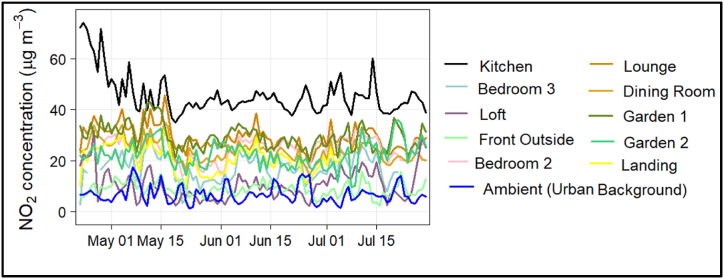
Daily mean time series of NO_2_ concentrations at multiple locations in a house property in Oxford.

Fig. 2 shows the daily mean NO_2_ time series at multiple indoor and outdoor locations across the study period. It was observed that the overall mean NO_2_ concentration in the kitchen was about 562 % higher than the background and 412.5 % higher than the front outdoor concentration. Notably, the daily mean NO_2_ levels in the kitchen exceeded the WHO daily average health-based guideline (25 μg m^−3^) for the entire duration of the sampling period, covering 100 % of the days (Table 3). Previous studies have identified that gas cooking hobs and boilers can be significant sources of various air pollutants such as CO, NO_x_ and PM [51]. Gas cooking appliances (including the one in this study) are not always mechanically vented to the outdoors and as a result, they are a potentially significant source of indoor air contamination as observed in our study. Similarly, Dėdelė and Miškinytė [52] also found that NO_2_ emissions indoors mainly depend on the type of stove used in the kitchen, highlighting that homes with gas stoves had significantly higher levels of NO_2_ in indoors compared with homes where electric stoves were used. Similar results were also found in a study by Kornartit, Sokhi [53], where NO_2_ levels were two times higher in kitchens with gas stoves than in kitchens with electric stoves.

***Impact of time-of-day and day-of-the-week factors on indoor NO***_***2***_***variations* –** The diurnal profile of NO_2_ concentrations captured morning and evening peaks in indoor locations (Figs. S4 and S5), which is in line with kitchen activities, as demonstrated in Table S1. The evening peaks in the kitchen were particularly significant compared to other indoor locations. Of particular note is that high levels in the kitchen corresponding to evening cooking remained elevated for several hours, only returning to ambient levels by the following morning, with the reduction at 7–8am likely corresponding to the kitchen door being opened. This NO_2_ associated with evening cooking also led to similar increases in concentrations at all of the other indoor sensors as NO_2_ dispersed through the house, particularly in locations closest to the kitchen such as the Dining Room and Lounge, although most of these other locations showed a return to ambient levels by around 9pm in the evening. Interestingly, the study found that NO_2_ concentrations at the property were almost constant throughout the weekdays and weekends; however, this may reflect consistent patterns of domestic activities due to the COVID-19 lockdown context. However, there was a clear decline in background (St Ebbe's) concentrations during weekends (Saturday and Sunday). The typical diurnal pattern of NO_2_ showed that the level of background concentration was correlated with traffic hours, indicating that traffic is a major contributor to outdoor NO_2_ pollution. This aligns with previous studies that found traffic as a prominent source of NO_2_ in Oxford [43,49].

### 3.3 Indoor/outdoor relationships and the role of indoor sources

**Fig. 3:**
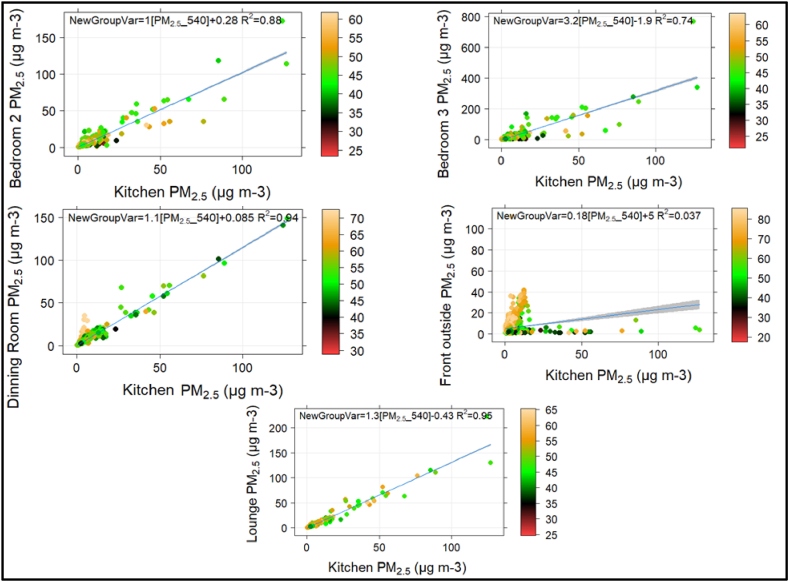
Scatter plots of Indoor locations against kitchen PM, coloured by relative humidity.

**Fig. 4:**
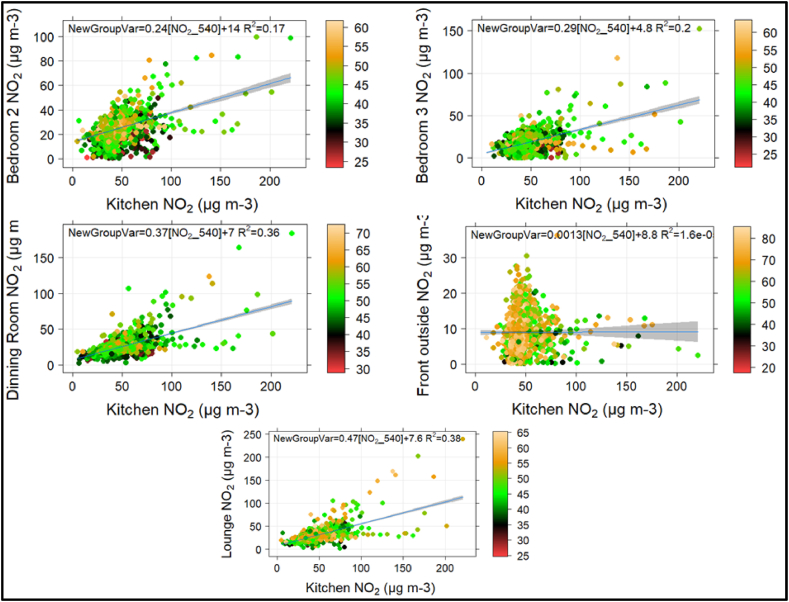
Scatter plots of Indoor locations against kitchen NO_2_ emissions, coloured by RH.

This section explores the contribution of outdoor PM to indoor air quality. To this end, scatter plots were generated to examine the relationship between indoor locations and ambient levels of PM_2.5_ and NO_2_, with relative humidity as a third variable (Fig. 3, Fig. 4, S6 and S7). We found no association between outdoor PM concentrations and indoor air quality (Fig. S6). It should be noted that outdoor PM_2.5_ levels were lower than long-term typical mean values in this setting during this period and these levels varied across the city in part because of the public health restrictions associated with COVID-19 [41]. However, there was a clear correlation between kitchen PM emissions and other indoor PM levels, suggesting that kitchen activities significantly affecting indoor air quality, as observed in the previous section (Fig. 3). Further, the scatter plots of indoor locations against ambient NO_2_ did not show a significant correlation (Fig. S7). However, we observed better correlations between kitchen and dining room concentrations, and between kitchen and lounge concentrations, suggesting that kitchen activities may have a more significant impact on indoor air quality than outdoor NO_2_ emissions (Fig. 4).

### 3.4 Indoor versus outdoor ratios (I/O)

**Fig. 5:**
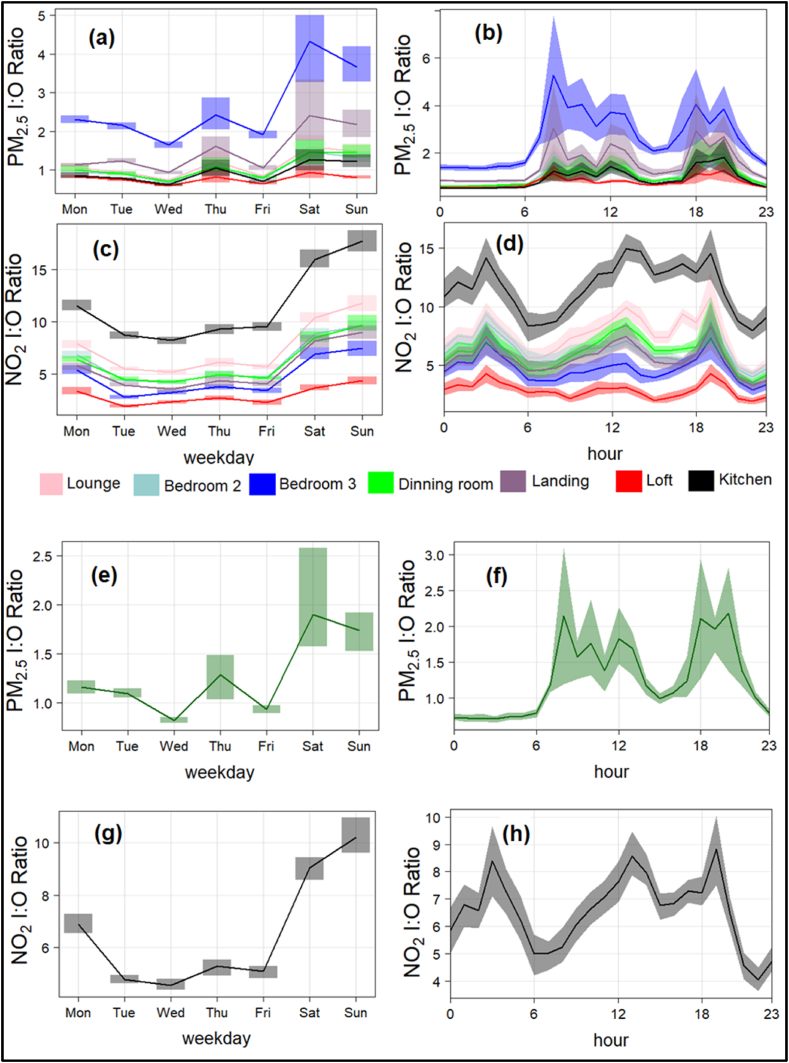
Diurnal patterns of day-of-week (**left panel**) and hourly (**right panel**) Indoor/Outdoor (I/O) ratios of PM_2.5_ and NO_2_ by individual rooms (**a – d**) and overall (**e – h).** The line shows the mean concentrations and shaded area as 95 % confidence interval in the difference in mean concentrations.

With respect to indoor concentration levels, the Indoor/Outdoor ratio (I/O) is a measure of the relative influence of outdoor air on levels of indoor air pollution. The ratio is calculated by dividing the indoor concentration by the corresponding front outdoor concentration. A value greater than 1 indicates that indoor sources are dominant. Overall, the mean I/O ratios for PM_2.5_ and NO_2_ were 1.28 and 6.54, respectively, with our PM_2.5_ ratio found to be similar to that reported in a study focused on London homes [54] (Table 4). Fig. 5 shows the mean I/O ratios for PM_2.5_ and NO_2_ by day-of-week and hourly, by indoor rooms (locations) and overall. Bedroom 3 had the highest I/O PM_2.5_ ratio, followed by the landing and kitchen, while the loft (which is rarely used) had the lowest ratio (Fig. 5 and Table 4). PM_2.5_ experienced peaks during early morning, afternoon, and evening, in line with household activities. Interestingly, higher I/O PM_2.5_ ratios were observed on weekends than weekdays due to increased household activities. The I/O ratio increased during peak times, suggesting that the major sources of indoor particles are those generated within the property rather than from dispersion of outdoor particles into the home [22]. Furthermore, the kitchen had the highest I/O NO_2_ ratios, while loft had the lowest. Distinct peaks were observed for I/O NO_2_ ratios compared to PM, with a night-time peak and afternoon and evening peaks. High I/O values of NO_2_ depend not only on indoor sources but also outdoor sources. The peak at 3–4 a.m. is due to lower outdoor NO_2_ concentrations (whereas the indoor value does not fall as swiftly due to presumed poorer indoor ventilation), while the increase in indoor NO_2_ in the afternoon and evening is due to cooking emissions. The difference between the peaks observed for PM and NO_2_ is not only due to differences in indoor sources but also due to outdoor concentrations and chemistry involved.

**Table 4:** Mean Indoor/Outdoor (I/O) ratios by individual rooms and overall, calculated from hourly averaged values.

I/O Ratios	PM_2.5_	NO_2_
**Kitchen (A)**	0.93	11.55
**Dining room (B)**	1.10	6.11
**Lounge (C)**	1.22	7.49
**Landing (D)**	1.51	5.57
**Loft (E)**	0.77	2.95
**Bedroom 2 (F)**	1.00	6.20
**Bedroom 3 (G)**	2.63	4.73
**Overall**	1.28	6.54

### 3.5 Influence of common domestic activities on NO_2_ and PM concentrations

**Fig. 6:**
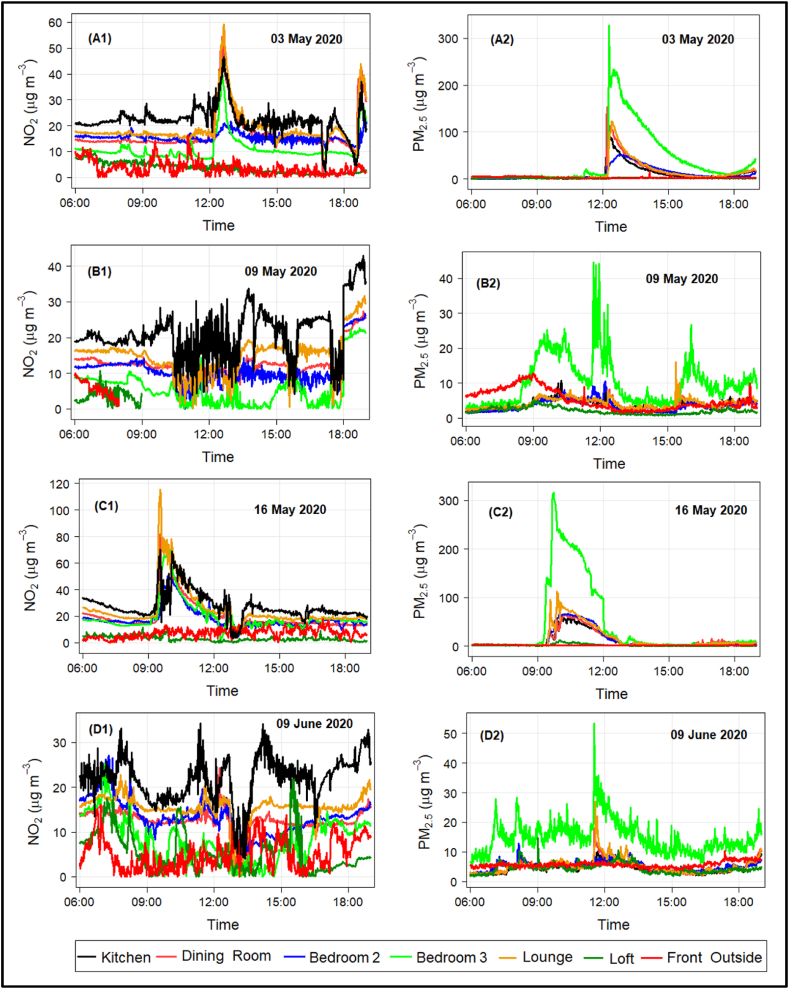
Concentrations of NO_2_ (**left panel**) and PM_2.5_ (**right panel**) on 1-mintute time resolution, from all sensors around specific domestic tasks. **Panel A:** Frying on a gas hob, **Panel B:** Vacuum cleaning, **Panel C:** Cooking on a gas hob with the smoke detector sounding, and **Panel D:** Printing.

As well as the long-term averages analysed in this study, it is of interest to examine the short-term increases in peak concentrations arising as a result of common domestic activities. Four specific examples are studied here: (i) frying on a gas hob, (ii) vacuum cleaning, (iii) frying on a gas hob with the smoke alarm sounding, and (iv) printing from a LaserJet printer, with the NO_2_ and PM_2.5_ concentrations before and after these events analysed (Fig. 6). The examples shown here are representative of when these activities took place throughout the study period (and data is available for researchers who wish to look at this in more detail). Cooking in an electric oven, which also took place regularly throughout the study duration resulted in no observable changes in NO_2_ or PM_2.5_ concentrations.

#### 3.5.1 Frying on a gas hob

Fig. 6A shows the NO_2_ (left) and PM_2.5_ concentrations arising from frying onions on the gas hob at 12:30 p.m. Both NO_2_ and PM_2.5_ concentrations can be seen to increase from the indoor sensors, outdoor sensors are unaffected. NO_2_ values roughly double and PM_2.5_ values increase by an even greater amount. The peak NO_2_ value is approximately 60 μg m^−3^ (below the UK's hourly mean limit value) and peak PM_2.5_ is over 300 μg m^−3^ in the unventilated Bedroom 3 and over 100 μg m^−3^ in the downstairs rooms (which are ventilated). The NO_2_ values have returned to normal levels within about 2 h whereas the PM_2.5_ values take around 4 h to return to normal levels.

#### 3.5.2 Vacuum cleaning (upstairs)

Fig. 6B shows the NO_2_ (left) and PM_2.5_ concentrations arising from vacuum cleaning in the upstairs part of the house at 12:00 noon. The NO_2_ concentrations are unaffected by the vacuum cleaning (as would be expected) as are the PM_2.5_ values. However, analysis of the PM_10_ data (not displayed) shows that a substantial increase in the values. This shows that the dust component is concentrated in that mass fraction and unlikely to be a source of the PM_2.5_ seen in the data.

#### 3.5.3 Frying on a gas hob with the smoke alarm sounding

Fig. 6C shows the NO_2_ (left) and PM_2.5_ concentrations arising from cooking pancakes on the gas hob at 09:15 a.m. and the smoke alarm in the house sounded at 09:30 with the downstairs door being open to ventilate the space until 10:00 a.m. Again, the indoor and outdoor sensors observe large increases in NO_2_ and PM_2.5_ concentrations, this time peaking at over 100 μg m^−3^ (NO_2_) and 300 μg m^−3^ (PM_2.5_) and on this occasion, elevated levels of pollutants remained until four (NO_2_) and five (PM_2.5_) hours after the cooking.

#### 3.5.4 Printing with a LaserJet printer

Fig. 6D shows the NO_2_ (left) and PM_2.5_ concentrations arising from printing a single sheet of paper using a LaserJet printer located in Bedroom 3 at 11:40 a.m. As expected, NO_2_ concentrations are unaffected by this, however PM_2.5_ increased notably – the 1-min peak being 50 μg m^−3^ – and indeed, looking at the PM_10_ values (again, not displayed), they peaked at over 1000 μg m^−3^. However, these high values were for a short time and normal levels resumed within 30 min.

## 4 Discussion

This study focuses on the temporal and spatial heterogeneity of indoor air pollution and emphasizes the significance of considering indoor environments and common domestic activities which generate high pollutant concentrations, in the context of the COVID-19 pandemic. It's important to note that individuals do not remain confined to a single space and utilize various rooms for diverse activities. Investigating these room-specific differences in pollutant concentrations, and how these change both hourly and daily, is a key strength of this study, providing a more comprehensive understanding of indoor air quality dynamics of relevance for health exposure studies. To the best of our knowledge this is the first study to obtain multiple continuous measurements of particle and gaseous pollutants for a ∼ 3-month period in a typical UK dwelling. The selected study household, a suburban 3-bedroom semi-detached residence inhabited by two people, is broadly typical of UK housing stock. In 2021, semi-detached homes were the prevalent housing type in England, comprising 30.6 % (7.6 million) of total dwellings [55]. In addition, we include a detailed assessment of domestic activities, alongside both indoor and outdoor air quality assessment, generating insights into pollutant sources and hazardous indoor air pollutant concentrations at levels which exceed WHO health-based guidelines. Notably, we identify high magnitude peak pollutant concentrations associated with typical domestic activities which are undertaken by many people across the UK on a daily basis (e.g. cooking, cleaning) as well as specific activities likely to be relevant to home-workers (e.g. printing), during a period of time when home-working levels were increased in the national population [56].

The findings of this study revealed significant variations in both PM_1_ and PM_2.5_ concentrations across different locations within the house property. The highest hourly concentrations were observed in an upstairs bedroom (used as a study), suggesting potential pollution sources in or near that area as well as (accurately) high utilisation. Cooking activities were identified as the major source of indoor particulate pollution, with peaks in PM concentrations corresponding to cooking events. Importantly, the study found that daily mean PM concentrations in Bedroom 3 (used as a study) exceeded (for 15 % of days from the study period) the recommended limits by the WHO air quality guidelines. Regarding NO_2_ pollution, higher concentrations were found indoors compared to outdoors, suggesting a dominant contribution of indoor sources. The kitchen, where gas cooking appliances were used, exhibited the highest levels of NO_2_. The emissions from the gas hob were identified as a key potential contributor to indoor NO_2_ pollution, particularly given no other combustion sources were present in the home.

This study also investigated the association between indoor and outdoor air quality, finding no significant correlation between indoor and outdoor PM concentrations. However, there was a clear correlation between kitchen PM emissions and indoor PM levels, indicating the substantial contribution of cooking activities on indoor PM air quality. Similarly, no significant correlation was found between indoor and outdoor NO_2_ concentrations; however, better correlations between NO_2_ levels in the kitchen and dining room, as well as between the kitchen and lounge, again reflect a greater influence of kitchen activities. Additionally, the high I/O ratios suggested that household sources (or activities) had a greater impact on indoor PM and NO_2_ levels compared to outdoor sources over the study period.

Overall, this study highlights the importance of indoor air pollution for exposure assessment studies, and the impact of specific sources, such as cooking activities, on peak indoor air pollution concentrations. Further, we demonstrate the utility of deploying multiple air quality sensors to provide valuable insights into spatial and temporal differences in indoor residential settings. The sensors operated throughout the study duration with no downtime and no noticeable intrusion on the occupants, who were able to lead their lives barely noticing the sensors (other than their visual presence).

Our findings suggest poor ventilation, particularly of upstairs rooms in the study household, led to increased pollutant concentrations following domestic activities. Importantly, these upstairs rooms are also close to sleeping areas where people spend a major amount of time (approximately one third of adult life) and therefore are relevant for targeted interventions reducing adverse health impacts of pollutant exposure. Although ventilation rates were not explicitly measured in this work qualitative observations can still be made and the findings suggest that inadequate ventilation may contribute, alongside increases due to human activities, to high concentrations in one upstairs domestic area (which did not have an opening window or any other means of external ventilation), which in well insulated homes – such as this study household – represents a key challenge for indoor air quality. Implementation of effective ventilation strategies to ensure adequate airflow and ventilation rates, facilitating the removal of pollutants and the introduction of fresh outdoor air. Mechanical ventilation systems, including exhaust fans and air purifiers, can be employed to achieve this goal. Furthermore, taking advantage of opportune outdoor air conditions, by opening windows and doors, can facilitate natural ventilation. Source control represents another pivotal approach, focusing on targeted mitigation of specific pollution sources. In a relatively well-insulated house with poor ventilation, indoor pollutant levels can accumulate. Encouraging the use of electric appliances in lieu of gas or wood-burning alternatives can significantly reduce emissions; or focusing retrofitting programmes to remove combustion appliances from homes may deliver indoor air quality benefits. Regular maintenance and thorough cleaning of ventilation systems, filters, and ducts are essential to prevent the accumulation of pollutants. Lastly, fostering awareness among occupants about the significance of optimal indoor air quality and providing educational resources on effective ventilation practices and pollutant reduction can empower individuals to actively maintain healthier indoor environments.

Behavioural changes regarding timing and method of cooking, informal ventilation (e.g., opening and closing windows and doors) are also important potential intervention options, as demonstrated by the peak levels generated by gas hob frying. Future research should investigate impacts of different cooking methods (e.g. boiling vs frying) on pollutant concentrations, alongside optimal timing of window and door opening when mechanical ventilation is limited or inadequate.

Despite the valuable insights provided by our study, there are some limitations that should be acknowledged. Firstly, the study was conducted during the COVID-19 lockdown period in 2020, which may not represent typical indoor (or outdoor) air quality conditions or occupation outside of a pandemic-related lockdown. The specific changes in residents' activities and behaviours during this period may have influenced the air quality patterns observed; however, many typical domestic activities such as cooking and cleaning are a key part of domestic life and could be expected to be undertaken irrespective of public health restrictions. Moreover, the study primarily examined PM and NO_2_ pollutants, but other pollutants that contribute to indoor air pollution, such as volatile organic compounds (VOCs) or formaldehyde, were not included in the analysis. Future studies could address these limitations to further enhance our knowledge of indoor air pollution dynamics.

## 5 Conclusions

This study investigated how domestic activities influence variations in indoor air pollutants over time and location within a single dwelling setting, aiming to inform public health strategies for improved indoor air quality management in the UK. In this work, air pollution concentrations (PM_1&2.5_ and NO_2_) were continuously measured at 10 second intervals at 7 indoor and 3 outdoor locations at a typical suburban property in Oxford, UK. The 3-month (100 day) study duration coincided with the first national lockdown reflecting COVID-19 public health restrictions implemented in the UK during spring 2020. The findings highlight significant variations in PM concentrations across different room locations, with cooking activities identified as the major source of indoor particulate pollution. During the study period, daily mean PM_2.5_ levels at indoor locations within the property exceeded the WHO 2021 Global Air Quality health-based guideline of 15 μg m^−3^ (daily average), on 1-15 days (1 %–15 %) (depending on location in the house) from the total period. The study also reveals higher levels of NO_2_ indoors, particularly in the kitchen where gas cooking appliances were used and in which the WHO Global Air Quality 24-hr Guideline was exceeded for every day of the entire study period. While no association was found between outdoor PM & NO_2_ levels and indoor concentrations, the stronger correlation between kitchen emissions and indoor pollutant levels, along with high I/O ratios, highlights the greater impact of household (particularly kitchen) activities on indoor air quality. The study emphasizes the role of indoor activities in contributing to indoor pollutant concentrations and highlights the need for targeted interventions to improve indoor air quality and achieve progress towards health-based guidelines. By implementing targeted interventions, such as reducing pollutant sources, improving ventilation systems, using effective extraction hoods, and promoting behavioural change towards cleaner cooking practices, it is possible to create healthier indoor environments and safeguard the well-being of occupants.

## Funding

The research was funded by the Natural Environment Research Council (NERC) [10.13039/100006147NE/V010360/1] and Natural Environment Research Council (NERC) [10.13039/100006147NE/V002449/1] This study/project is also funded by the National Institute for Health Research (NIHR) [10.13039/501100001921PHR 130095]. The views expressed are those of the author(s) and not necessarily those of the NIHR or the Department of Health and Social Care.

## Ethics statement

### Institutional review board statement

Ethical approval for this study was provided on January 5, 2021 by the University of Birmingham Science, Technology, Engineering and Mathematics Ethical Review Committee. ERN_20–0831.

## Data availability statement

The data supporting the findings of this study have been deposited at the 10.13039/501100000769Oxford University Research Archive (ORA) and are openly available at the following URL/DOI: https://doi.org/10.5287/ora-dodq8yx5b.

## CRediT authorship contribution statement

**Ajit Singh:** Writing – original draft, Visualization, Validation, Methodology, Investigation, Formal analysis, Data curation, Conceptualization. **Suzanne E. Bartington:** Writing – review & editing, Supervision, Methodology, Funding acquisition. **Pedro Abreu:** Writing – review & editing. **Ruth Anderson:** Writing – review & editing. **Nicole Cowell:** Writing – review & editing. **Felix C.P. Leach:** Writing – original draft, Visualization, Supervision, Investigation, Funding acquisition, Data curation, Conceptualization.

## Declaration of competing interest

The authors declare that they have no known competing financial interests or personal relationships that could have appeared to influence the work reported in this paper.
