# Supplementary material for: Impacts of daily household activities on indoor particulate and NO2 concentrations; a case study from oxford UK
Source: Heliyon. 2024 Jul 5;10(15):e34210. doi: 10.1016/j.heliyon.2024.e34210 (PMC11333897; doi:10.1016/j.heliyon.2024.e34210)
Supplement: Multimedia component 1 [file mmc1.docx]

*Supplementary*

Impacts of daily household activities on indoor particulate and NO_2_ concentrations; a case study from Oxford UK

Ajit Singh^a, b *^, Suzanne E. Bartington^a^, Pedro Abreu^c^, Ruth Anderson^d^, Nicole Cowell^b^, Felix C.P. Leach^e *^,

**
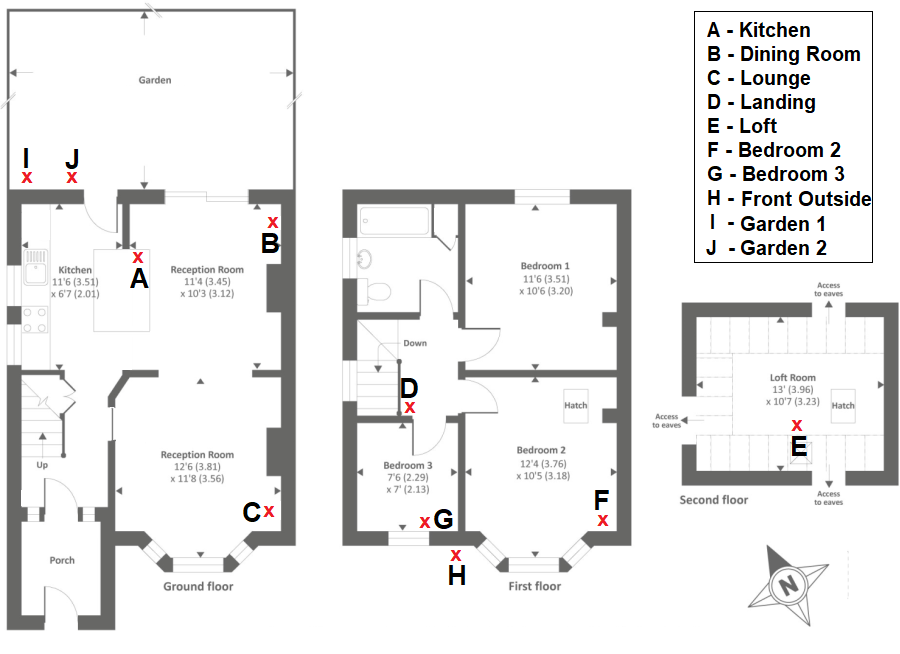
**

**Figure S1** Floor plan of the study house with the air quality sensor locations indicated by the A – J.

**Table S1** Recorded entries from the domestic activity log (in GMT)

| **Date** | **Time** | **Activity** | **Notable** |
| --- | --- | --- | --- |
| 22-04-2020 | 18:48 pm | Cooking in electric oven |  |
| 23-04-2020 | 18:58 pm | Cooking on gas hob   - Boiling pots - Frying oil/chicken |  |
| 24-04-2020 | 07:00 am | Toaster |  |
| 24-04-2020 | 07:50 am | Toaster |  |
| 24-04-2020 | 12:30 pm | Kettle |  |
| 24-04-2020 | 19:00 pm | Cooking in electric oven (door was open) |  |
| 25-04-2020 | 8:25 am | Cooking in electric oven |  |
| 25-04-2020 | 16:00 pm | Baking in electric oven |  |
| 25-04-2020 | 18:30 pm | - Water boiling on gas hob - Grill (slightly burned bread at 20:08 pm and door open) |  |
| 26-04-2020 | 12:30 pm | Frying on gas hob | door open |
| 26-04-2020 | 17:15 pm | - Cooking on electric oven - Boiling water on gas hob - Three candles burning |  |
| 27-04-2020 | 19:30 pm | Boiling water on gas hob |  |
| 28-04-2020 | 18:30 pm | Frying on gas hob |  |
| 29-04-2020 | 12:30 pm | - Hoovering (downstairs) - Toaster |  |
| 29-04-2020 | 17:30 pm | - Hoovering (upstairs) |  |
| 29-04-2020 | 18:30 pm | Cooking in electric oven |  |
| 30-04-2020 | 18:20 pm | Cooking in electric oven |  |
| 01-05-2020 | 19:00 pm | Cooking in electric oven |  |
| 02-05-2020 | 08:00 am | Cooking in electric oven |  |
| 02-05-2020 | 19:20 pm | Cooking in electric oven |  |
| 03-05-2020 | 12:30 pm | Frying (onion) on gas hob |  |
| 03-05-2020 | 17:45 pm | Cooking in electric oven |  |
| 03-05-2020 | 18:30 pm | Cooking on gas hob |  |
| 03-05-2020 | 19:20 pm | Candle burning |  |
| 04-05-2020 | 12:00 pm | Boiling water on gas hob |  |
| 05-05-2020 | 20:00 pm | Cooking in electric oven |  |
| 06-05-2020 | 19:20 pm | Cooking in electric oven |  |
| 07-05-2020 | 12:00 pm | Boiling water on gas hob |  |
| 07-05-2020 | 19:00 pm | Cooking in electric oven |  |
| 09-05-2020 | 09:30 am | Cooking in electric oven |  |
| 09-05-2020 | 12:00 pm | Hoovering (upstairs) |  |
| 09-05-2020 | 20:00 pm | Cooking in electric oven |  |
| 10-05-2020 | 13:00 pm | Cooking in electric oven |  |
| 10-05-2020 | 18:45 pm | - Cooking in electric oven - Cooking on gas hob |  |
| 12-05-2020 | 14:30 pm | Furniture polish (upstairs) |  |
| 12-05-2020 | 12:30 pm | Hoovering (upstairs) |  |
| 12-05-2020 | 18:00pm | Cooking in electric oven |  |
| 12-05-2020 | 20:50 pm | - Cooking on gas hob - Boiling and stir fry - Two candles burning |  |
| 13-05-2020 | 12:00 pm | Cooking on gas stove (egg scrambling) |  |
| 13-05-2020 | 19:00 pm | - Cooking on gas hob - Cooking in electric oven |  |
| 14-05-2020 | 12:15 pm | Boiling on gas hob |  |
| 14-05-2020 | 19:30 pm | Cooking in electric oven |  |
| 15-05-2020 | 19:10 pm | Cooking in electric oven |  |
| 16-05-2020 | 09:15 am | - Cooking on gas hob (pancakes) | Smoke alarm at 09:30 am, door opened till 10:00 am |
| 16-05-2020 | 12:15 pm | Boiling on gas hob |  |
| 16-05-2020 | 18:45 pm | Cooking in electric oven |  |
| 17-05-2020 | 08:45 am | Frying on gas hob (bacon) |  |
| 17-05-2020 | 12:30 pm | - Boiling on gas hob - Frying on gas hob |  |
| 17-05-2020 | 20:00 pm | Cooking in electric oven |  |
| 18-05-2020 |  |  |  |
| 19-05-2020 | 19:30 pm | Cooking in electric oven |  |
| 20-05-2020 | 19:00 pm | Cooking in electric oven |  |
| 21-05-2020 | 19:00 pm | Cooking on gas hob |  |
| 22-05-2020 | 19:15 pm | Cooking in electric oven |  |
| 23-05-2020 | 21:30 pm | - Cooking in electric oven - Frying on gas hob | Door open |
| 23-05-2020 | 19:00 pm | Cooking in electric oven |  |
| 24-05-2020 | 09:20 am | Cooking in electric oven |  |
| 31-05-20201 | 12:30 pm | - Cooking in electric oven - Frying and boiling on gas hob |  |
| 31-05-2020 | 19:00 pm | Cooking in electric oven |  |
| 02-06-2020 | 19:00 pm | Cooking in electric oven |  |
| 03-06-2020 | 12:00 pm | Boiling on gas hob |  |
| 03-06-2020 | 19:00 pm | Cooking in electric oven |  |
| 05-06-2020 | 19:00 pm | Cooking in electric oven |  |
| 06-06-2020 | 08:00 am | Frying on gas hob (bacon) | Smoke alarm |

***Definitions of the diverse activities documented in the activity log are as follows:**

***Cooking (or Baking) in electric oven:*** *Any use of the electric oven for baking, roasting, or cooking dishes.*

***Cooking on gas hob:*** *Activities involving the use of the gas hob for stovetop cooking.*

***Frying on gas hob:*** *Activities involving the use of the gas hob for frying food items.*

***Boiling on gas hob:*** *Engaging in cooking methods that involve boiling food items in water using the gas hob.*

***Toaster:*** *Operation of the toaster for toasting bread or other food items.*

***Furniture polish:*** *Application of furniture polish or cleaning agents.*

***Candle burning:*** *Lighting and burning of candles for ambience or scent.*

***Hoovering:*** *Operating a vacuum cleaner for cleaning activities.*

*­
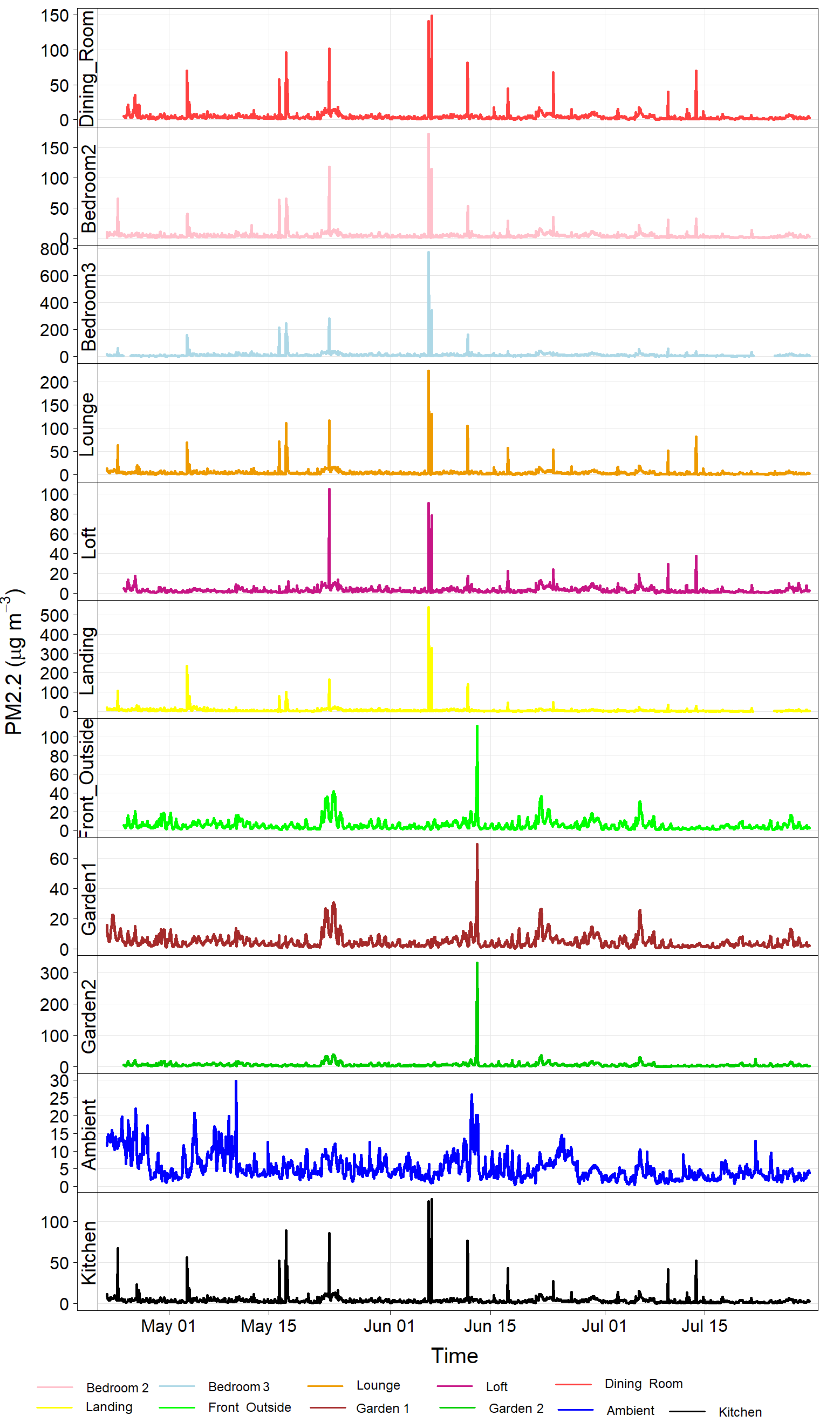
*

**Figure S2** Hourly variations of PM_2.5_ concentrations observed at multiple locations within the study property

**
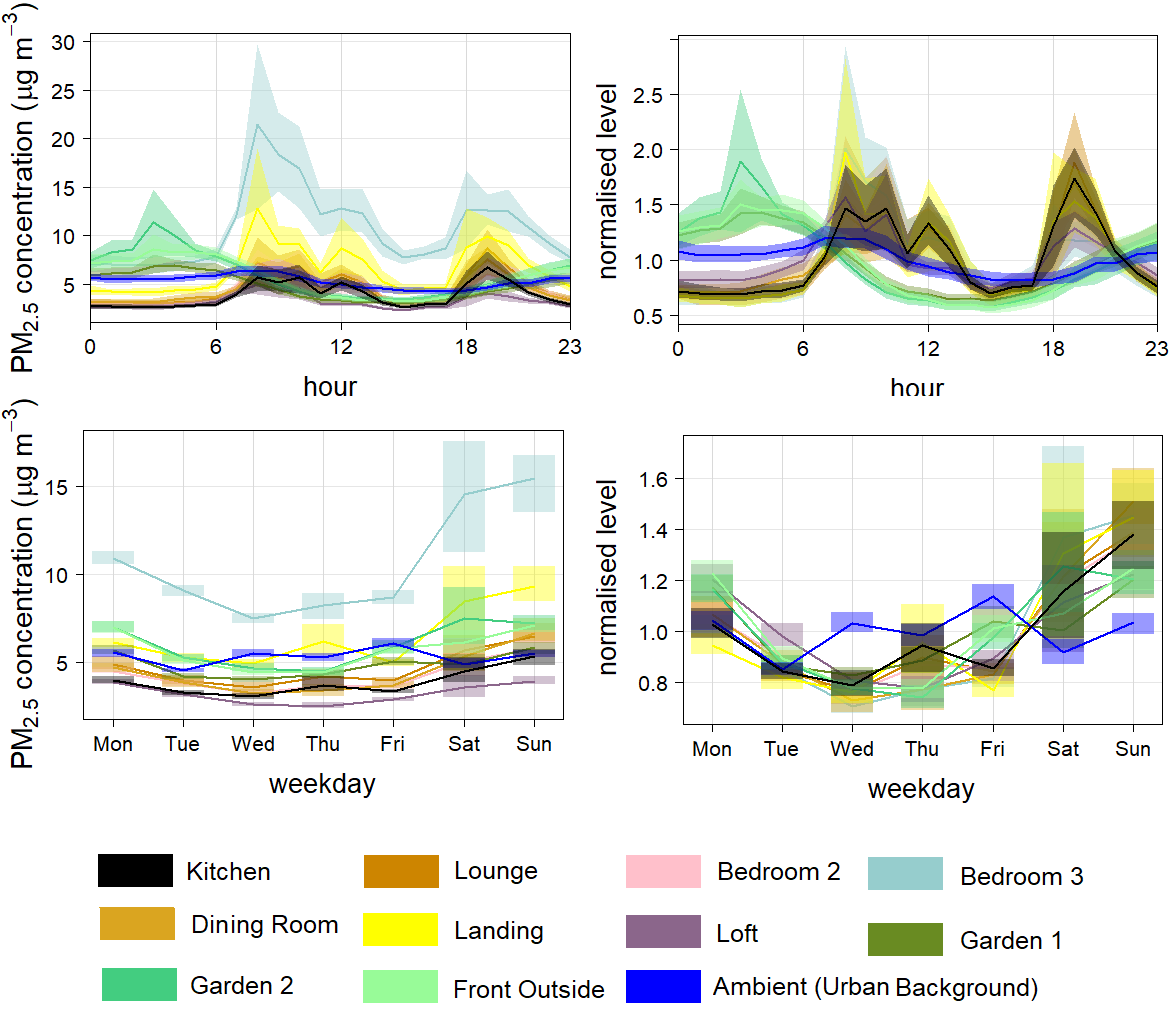
Figure S3** Hourly mean **(left panel)** and normalized (**right panel**) to mean diurnal and day-of-week profiles of PM_2.5_ concentrations, where shading shows the 95% confidence interval with respect to overall mean.

**
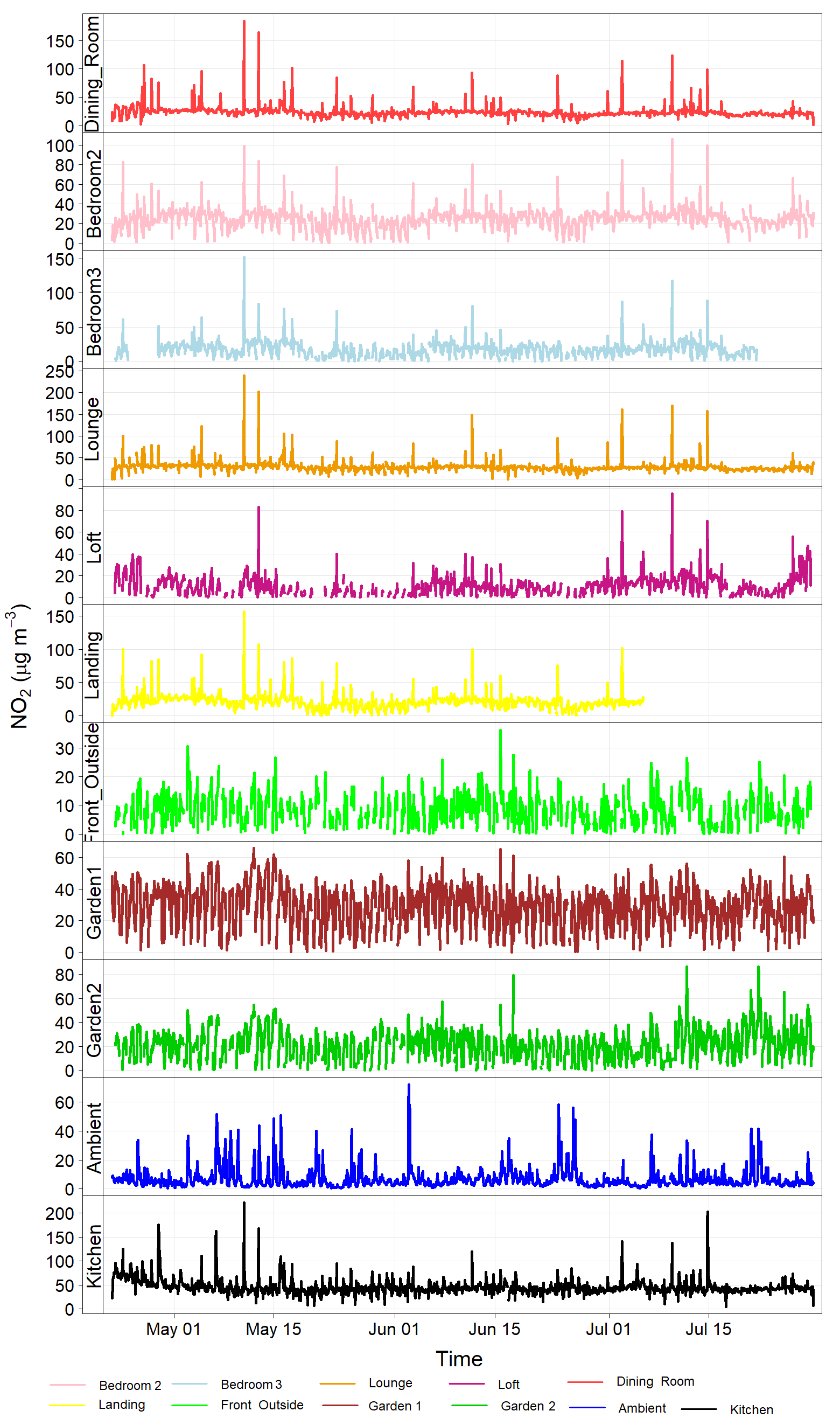
**

**Figure S4** Hourly mean time-series of NO_2_ concentrations at multiple locations in a house property in Oxford.

**
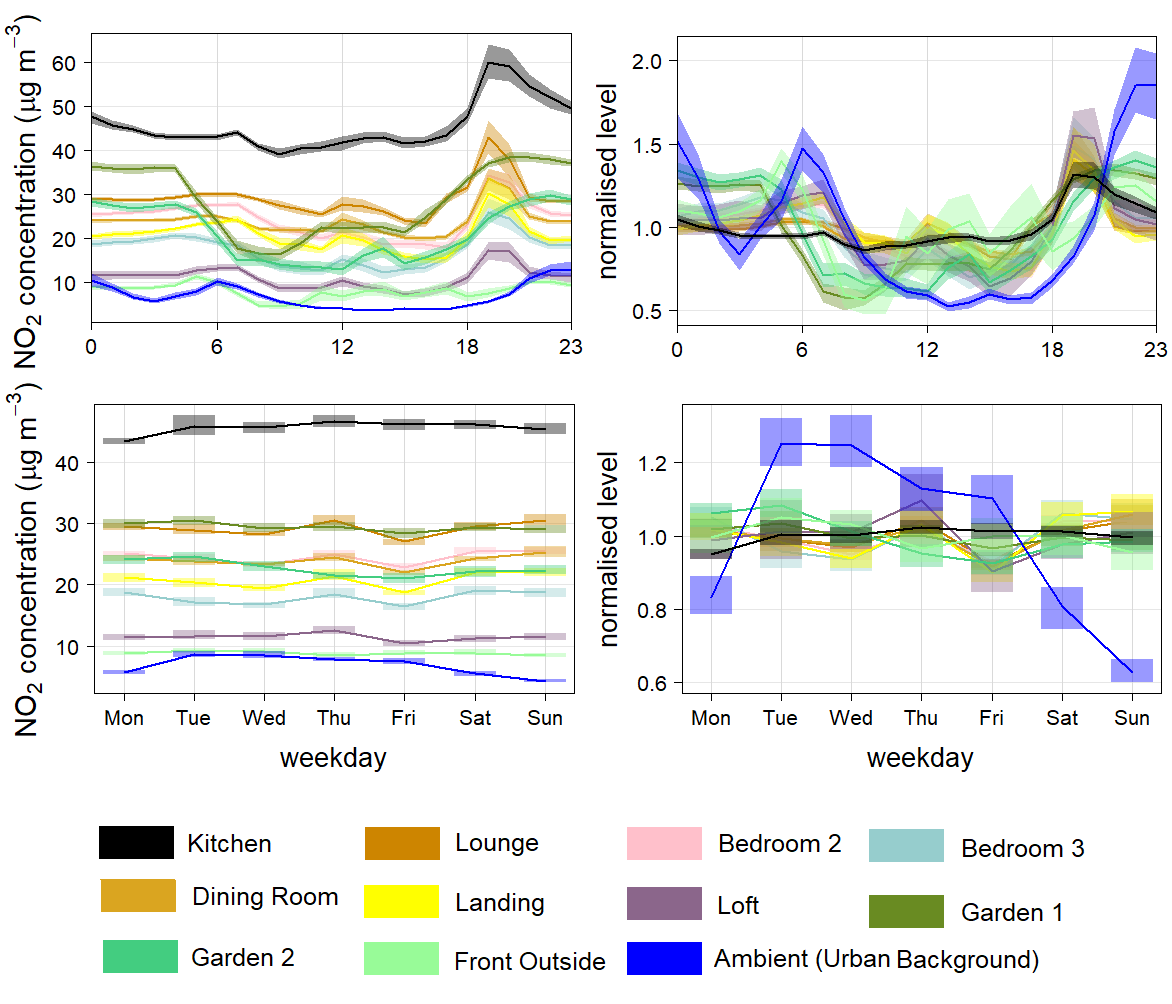
Figure S5** Hourly mean **(left panel)** and normalized (**right panel**) diurnal and day-of-week profiles of NO_2_ concentrations, where shading shows the 95% confidence interval with respect to overall mean.

**
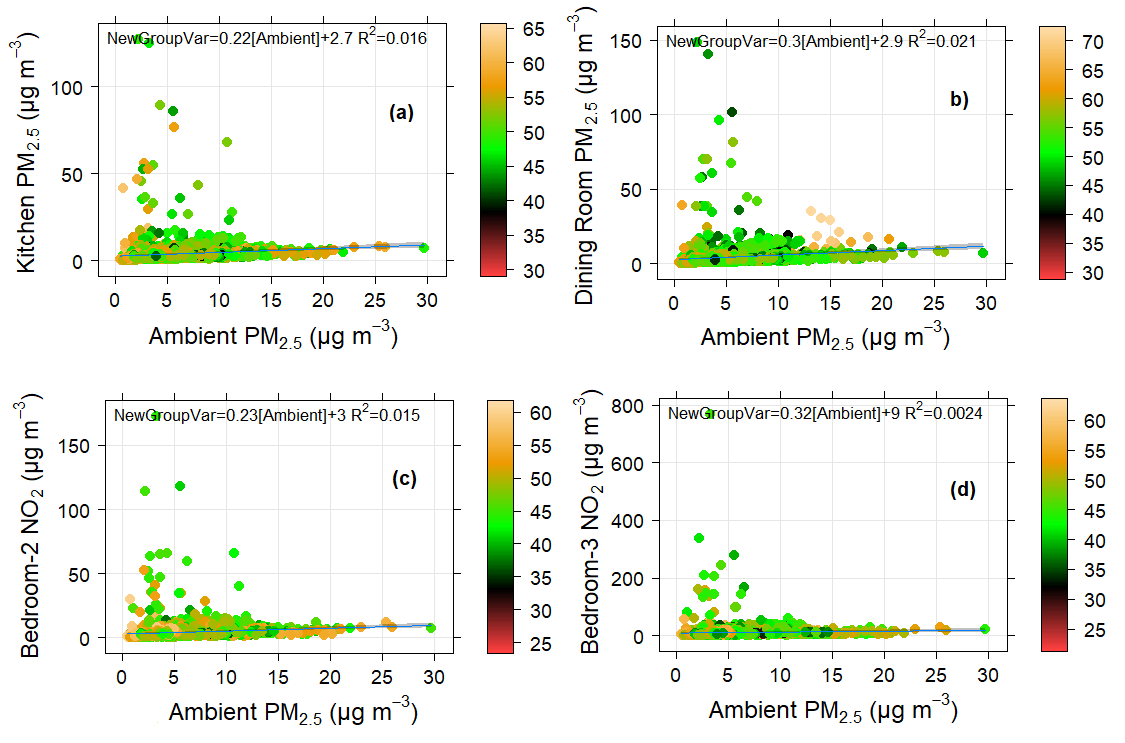
Figure S6** Scatter plots of indoor PM against ambient (outdoor) PM concentrations, coloured by relative humidity.

**
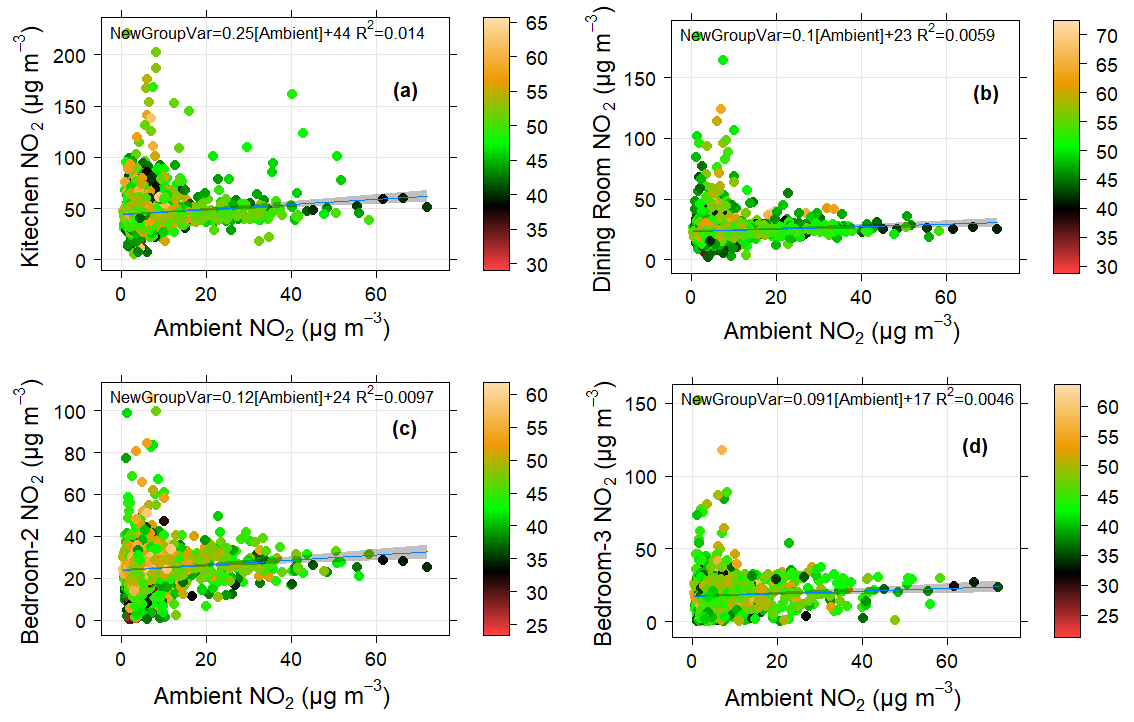
Figure S7** Scatter plots of Indoor NO_2_ against outdoor NO_2_ emissions, coloured by relative humidity.
